# Identification and Analysis of Novel Biomarkers Involved in Chromophobe Renal Cell Carcinoma by Integrated Bioinformatics Analyses

**DOI:** 10.1155/2020/2671281

**Published:** 2020-02-07

**Authors:** Wei Zhang, Yin Xu, Jinghan Zhang, Jun Wu

**Affiliations:** ^1^Department of Human Anatomy, Kangda College, Nanjing Medical University, Lianyungang, Jiangsu, China; ^2^Neonatal Medical Center, Children's Hospital of Nanjing Medical University, Nanjing, Jiangsu, China; ^3^The Research Center for Bone and Stem Cells, Department of Anatomy, Histology and Embryology, Nanjing Medical University, Nanjing, Jiangsu, China

## Abstract

In renal cell carcinoma, chromophobe renal cell carcinoma (ChRCC) is a distinct subtype, whose clinical manifestations often lack specificity, and the molecular mechanisms of ChRCC tumorigenesis remain generally vague. The target of this study was to discover novel biomarkers involved in ChRCC by integrated bioinformatics analyses. We found 2608 differentially expressed genes (DEGs), of which 1518 were upregulated and 1090 were downregulated. Gene ontology (GO) analysis of DEGs uncovered significant functional enrichment in three aspects: biological process (BP), molecular function (MF), and cellular component (CC). The results of Kyoto Encyclopedia of Genes and Genomes (KEGG) enrichment analysis indicated DEGs were largely enriched in retinol metabolism, arachidonic acid metabolism, and pentose and glucuronate interconversions. Then, the protein–protein interactions (PPI) network was constructed and top three hub genes were identified by the Cytoscape plugin cytoHubba. Through calculating the degree, betweenness centrality, and Stress of mRNAs, CENPA was upregulated and KNG1 and AGT were downregulated. A survival assay performed according to Oncomine data showed only CENPA high expression exhibited a worse prognosis. This study identified crucial genes and pathways for the progress of ChRCC, and CENPA might be a novel biomarker for diagnosis, treatment, and prognosis of ChRCC.

## 1. Introduction

Chromophobe renal cell carcinoma (ChRCC), derived from distal convoluted tubules and cortical collecting conduits, could be a distinct subtype of renal cell carcinoma, which accounts for almost 5% of renal cell carcinoma subtypes [1]. Although ChRCC is relatively inert, once metastasized, ChRCC patients have the same survival rate as metastatic clear cell renal cell carcinoma (ccRCC) [2]. The clinical manifestations of ChRCC often lack specificity, which makes it difficult for early diagnosis [3, 4]. Thus, understanding in-depth pathogenesis of ChRCC is urgently demanded for early diagnosis, treatment, and prognosis.

The molecular mechanisms of ChRCC tumorigenesis remain generally unclear. Many researchers through retrospective analysis found some related markers to ChRCC, such as c-Met [5], PD-L2 [6], and the oncogene KIT expression [7], which were almost upregulated, associated with metastatic progression and poor survival in ChRCC. Genetically, ChRCC are known to have different forms of chromosomal anomalies. ChRCC often involves in gains in chromosomes 4, 7, 11, 12, 14q, and 18q, as well as losses in chromosomes Y, 1, 2, 6, 10, 13, 17, and 21, which may result in tumor suppressor gene inactivation and promote tumorigenesis [8, 9]. Changes in the number of chromosomes are important features of human cancer and may reflect potential genomic instability [10], which might lead to tumor suppressor gene mutation or deletion, such as PTEN, p53 [11], RB1, and ERBB4 [12], promoting tumorigenesis and distant metastasis. Thus far, all of these genomic findings have been completed in retrospective studies using archived tumor samples and deserve further validation.

At present, with the wide application of sequencing, bioinformatics analyses have great advantage for understanding the pathophysiological mechanisms of ChRCC. Wang et al. have instituted CFTR as a key gene based on the GEO database [13]. No other records have been found to study the disease using this method. In order to analyze the cancer more accurately, in our study, we integrated the TCGA database, using bioinformatics analyses to explore likely molecular mechanisms and novel biomarkers in ChRCC and identify CENPA was a vital gene involved in ChRCC.

## 2. Materials and Methods

### 2.1. Identification of DEGs from TCGA Database

The TCGA database contains exhaustive, multidimensional maps of key cancer genome changes in various cancers [14], which was selected for our study. All data have been collected and analyzed by the R language. Samples were then subjected to differential expression analysis using the edgeR package. Genes with log fold-change (FC)| > 2 and *P* < 0.05 were considered to be DEGs.

### 2.2. GO and KEGG Pathway Analysis

On the basis of DEGs, gene ontology (GO) and Kyoto Encyclopedia of Genes and Genomes (KEGG) pathway enrichment analyses were performed by using the R package clusterProfiler [15]. GO enrichment analysis is analyzed in three aspects: biological process (BP), molecular function (MF), and cellular component (CC). The KEGG database stores a wealth of information about genomes, biological pathways, chemicals and drugs, and diseases, which is widely used to identify functional and metabolic pathways associated with the overlapping DEGs [16]. A *P*-value of was <0.05 identified as significant difference.

### 2.3. PPI Network and Relative Expression of Hub Genes

The PPI network for screening genes was established through the STRING online database, and an interaction score of ≥0.4 was set. Then, the results were visualized by the Cytoscape software (version 3.6.1, http://www.cytoscape.org/). The hub genes by calculating the degree, betweenness centrality, and Stress were screened in CytoHubba. To further validate the mRNA levels in ChRCC, we examined the relative expression of the hub genes in Oncomine, which is an online platform that provides cancer microarray datasets and data exploration capabilities to validate the expression of specific genes in a variety of cancers, thereby helping discover the potential genes involved in tumorigenesis and progression. *P* < 0.05 represented a statistically significant threshold.

### 2.4. Survival Analysis of Hub Genes

For hub genes that were significantly associated with survival, the relationship between mRNA expression level and overall survival were estimated using the online tool UALCAN (http://ualcan.path.uab.edu), which is a convenient, interactive web resource for analyzing cancer transcriptome data based on the Oncomine dataset [17].

## 3. Results

### 3.1. Identification of DEGs in ChRCC

**Figure 1:**
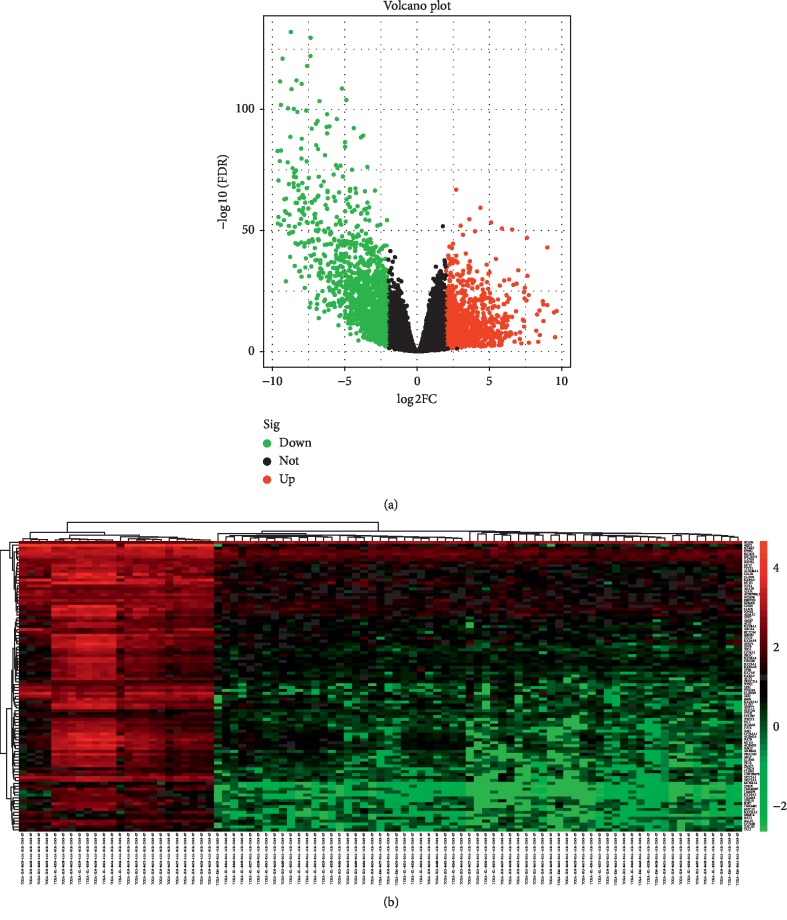
Identification of DEGs in the TCGA database. (a) Volcano plot of DEGs between ChRCC and normal control. (b) The expression heatmap of top 100 DEGs. Red or green dots represent upregulated or downregulated genes, respectively. Genes without any significant difference are in black. The differences are set as *P* < 0.05 and |log(FC)| > 2. DEGs, differentially expressed genes; ChRCC, chromophobe renal cell carcinoma.

After performing integrated analysis between tumor and normal tissues from the TCGA database, a total of 2608 DEGs were found. Among them, 1518 were upregulated and 1090 were markedly downregulated (*P* < 0.05 and |log(FC)| > 2) The DEGs from the dataset are shown in Figure 1(a). Red or green dots represent upregulated or downregulated genes, respectively. The top 100 DEGs were displayed through the heat map (Figure 1(b)).

### 3.2. GO and KEGG Pathway Enrichment Analyses of These DEGs

**Figure 2:**
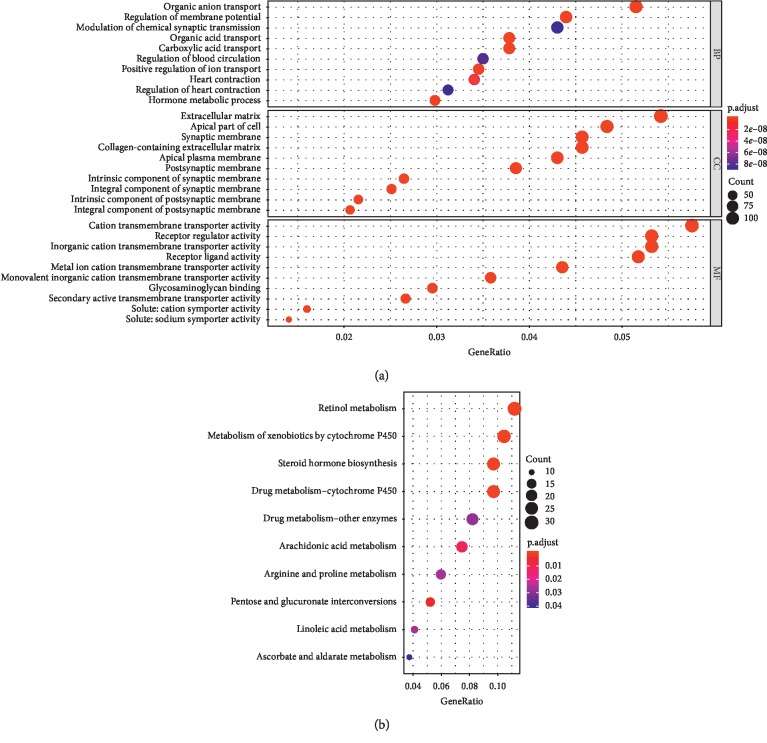
Functional and pathway enrichment analyses of DEGs in ChRCC. (a) Top ten of GO analysis. GO, gene ontology; BP, biological process; MF, molecular function; CC, cellular component. (b) Top ten of KEGG pathway enrichment. KEGG, Kyoto Encyclopedia of Genes and Genomes.

For better understanding of the DEGs, GO analysis was performed in Figure 2(a) and divided into biological process (BP), cellular component (CC), and molecular function (MF). The main biological processes that the DEGs were enriched in are presented, including organic anion transport, regulation of membrane potential, and organic acid transport. For the cellular component, the DEGs were particularly enriched in the apical plasma membrane, apical part of the cell and extracellular matrix. According molecular function, DEGs were significantly enriched in receptor ligand activity, receptor regulator activity, and cation transmembrane transporter activity. Then, the KEGG pathways program was used to reveal the critical pathway, in which a total of 10 pathways were identified, such as retinol metabolism, arachidonic acid metabolism, and pentose and glucuronate interconversions (Figure 2(b)).

### 3.3. PPI Network and Hub Genes Identification

**Figure 3:**
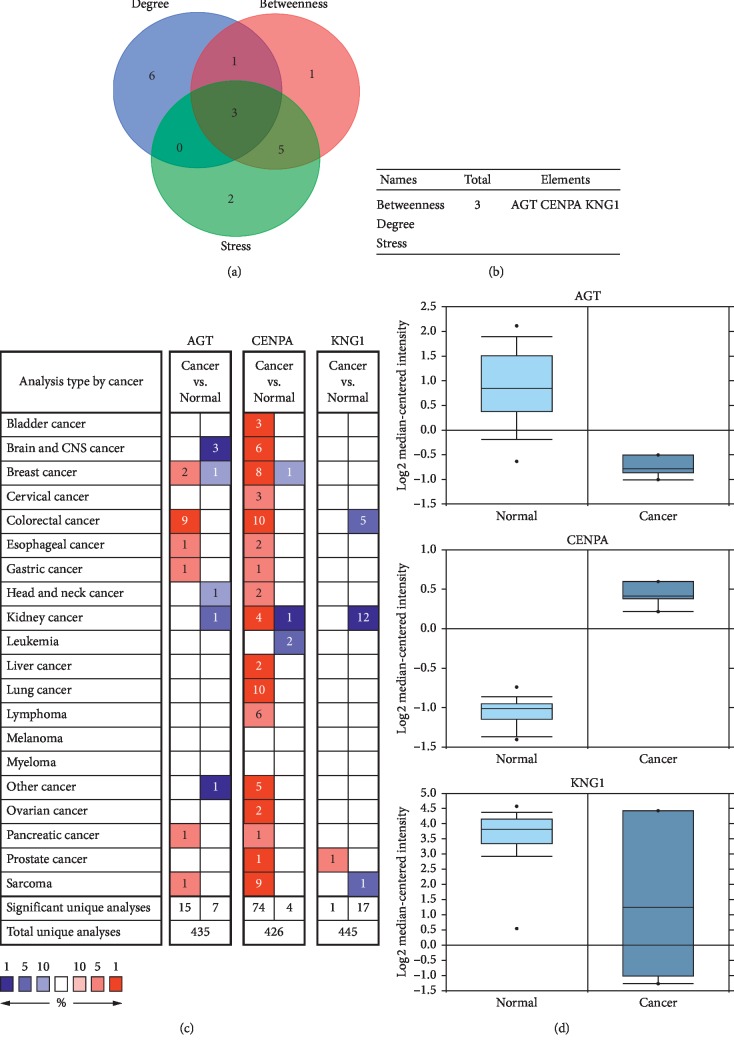
Identification and relative expression of hub genes in ChRCC. (a, b) Three hub genes were selected by overlapping the top ten genes based on three ranked methods, including the degree, betweenness centrality, and Stress. (c) An overview of mRNA levels of hub genes in a variety of cancers based on Oncomine. The numbers in colored cells show the quantities of datasets with statistically significant mRNA overexpression (red) or underexpression (blue) of target genes. Cell color was determined by the best gene rank percentile for the analysis within the cells. The threshold was set as *P* < 0.05. (d) Relative expression of hub genes between ChRCC and normal samples. The threshold was set as *P* < 0.05.

In order to discover the potential association between these DEGs, a PPI network of DEGs was established in STRING database ([Supplementary-material supplementary-material-1]). Top three hub genes were selected by calculating the degree, betweenness centrality, and Stress of Cytoscape plugin cytoHubba (Figures 3(a) and 3(b)). Then, an overview of mRNA levels of hub genes in a variety of cancers based on Oncomine is presented in Figure 3(c). As shown in Figure 3(d), one upregulated gene was CENPA, and two downregulated genes were KNG1 and AGT.

### 3.4. Survival Analysis of Hub Genes

**Figure 4:**
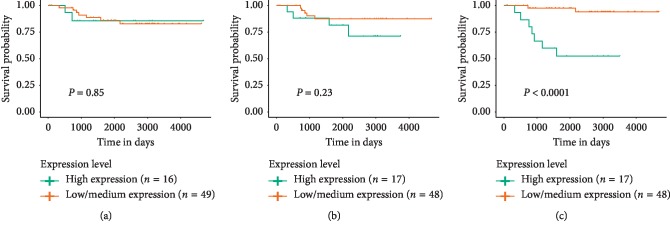
Prognostic value of hub genes for the overall survival of patients with ChRCC. (a) Effect of KNG1 expression level on KICH patient survival. (b) Effect of AGT expression level on KICH patient survival. (c) Effect of CENPA expression level on KICH patient survival. Patients were divided into two groups including low-expression and high-expression groups according to the median gene expression. *P* < 0.05 was considered as significant. ChRCC, chromophobe renal cell carcinoma; KICH, chromophobe renal cell carcinoma.

To further research the survival value of hub genes in ChRCC, this study performed a survival assay according to Oncomine data. As shown in Figure 4, only relatively high expression of CENPA was associated with worse prognosis of ChRCC patients (*P* < 0.05), while expression of KNG1 or AGT had no statistically significant effect on patients' overall survival. Thus, CENPA may serve as a potential and novel biomarker for ChRCC.

## 4. Discussion

Although ChRCC usually appears as a larger tumor, it is relatively inert [18], and about 5%–10% of patients eventually develop metastases [4, 19], thus increasing the mortality rate of ChRCC. At present, the molecular mechanisms of ChRCC tumorigenesis remain generally vague. Therefore, the etiology and molecular mechanisms of ChRCC are found to be critical for cancer treatment and prevention. The application of chips and bioinformatics has been widely used to discover DEGs in tumorigenesis, diagnosis, and treatment.

In our study, DEGs were firstly screened from the TCGA database in tumor and normal samples, and then GO and KEGG pathway analyses were performed. A PPI network was established, and top three hub genes were identified by cytoHubba, which include an upregulated gene (CENPA) and downregulated genes (KNG1, AGT). Then, survival analysis showed upregulation of CENPA was associated with lower overall survival of ChRCC patients, while expression of KNG1 or AGT had no statistical influence.

Studies have shown that kininogen-1 (KNG1) could suppress angiogenesis [20] and metastasis [21]. KNG1 was studied as the core gene and downregulated in the glioma cells [22], which was also identified as a serum biomarker for colorectal cancer [23]. Overexpression of KNG1 could inhibit cell viability and angiogenesis and promote the apoptosis and G1 phase cell cycle arrest of glioma cells [22]. In the present study, KNG1 was downregulated in ChRCC, but there was no statistical influence on survival. Thus, the relationship between this gene and the tumor progression needs further verification. Angiotensinogen (AGT) is one of the significant parts of the renin–angiotensin system (RAS), widely known as a blood pressure regulation system [24]. Decades ago researchers have implicated AGT with an inhibition of human endothelial cell proliferation, cell migration, and angiogenesis in vitro [25, 26]. In breast cancer risk, AGT was involved in postmenopausal women [27], and the pro-tumor properties of high glucose in breast cancer cells are mainly attributed to inhibition of AGT [28]. However, an article pointed out AGT was overexpressed in lung adenocarcinoma tissue [29]. Moreover, the association between polymorphisms in the AGT and lung cancer risk showed no consistent results [30]. So, the association of AGT with cancer risk has still been inconsistent. In our findings, AGT was downregulated but was not associated with the prognosis.

Centromere protein-A (CENPA) is a histone-H3 variant that regulates cell division and has been associated with cancer progression [31]. CENPA was highly expressed in epithelial ovarian cancer [32], breast cancer [33], osteosarcoma [34], and lung adenocarcinoma [35]. Increase in CENPA by immunohistochemical analysis in breast cancer samples trended towards an adverse outcome [8]. In hepatocellular carcinoma patients, CSN5 depletion took effective effects through downregulation of SMAD5-related pathways including CENPA, which represented a potential target for therapeutic approaches [36]. However, the pathological and clinical roles of CENPA in ChRCC remain unclear. Our study has identified CENPA was a key gene and upregulated in ChRCC patients control with normal patients. In addition, survival analysis results suggested that CENPA may be a prognostic indicator for patients with ChRCC.

Finally, based on those analyses, DEGs were identified and CENPA could be a novel biomarker for early diagnosis, treatment, and prognosis and might play an important role in ChRCC progression. Further comprehensive and in-depth research on this gene will be very valuable.

## 5. Conclusions

In this study, we conducted integrated bioinformatics analyses, which consist of identification of DEGs, GO and KEGG enrichment analyses, a PPI network, identification of hub genes, and survival analysis, to suggest potential DEGs for progression of ChRCC. Moreover, only the hub gene CENPA is related to overall survival, which may be a novel biomarker involved in chromophobe renal cell carcinoma. In the future, the biological functions of these novel genes and the potential pathogenesis of ChRCC still need to be further explored.

## Data Availability

The data used to support the findings of this study are included within the article.

## Supplementary Materials

Supplementary Materials The PPI network for screening genes was established through the STRING online database.
