# Supplementary material for: Identification and Analysis of Novel Biomarkers Involved in Chromophobe Renal Cell Carcinoma by Integrated Bioinformatics Analyses
Source: Biomed Res Int. 2020 Feb 7;2020:2671281. doi: 10.1155/2020/2671281 (PMC7029304; doi:10.1155/2020/2671281)
Supplement: Supplementary Materials — The PPI network for screening genes was established through the STRING online database. [file 2671281.f1.pdf]

A

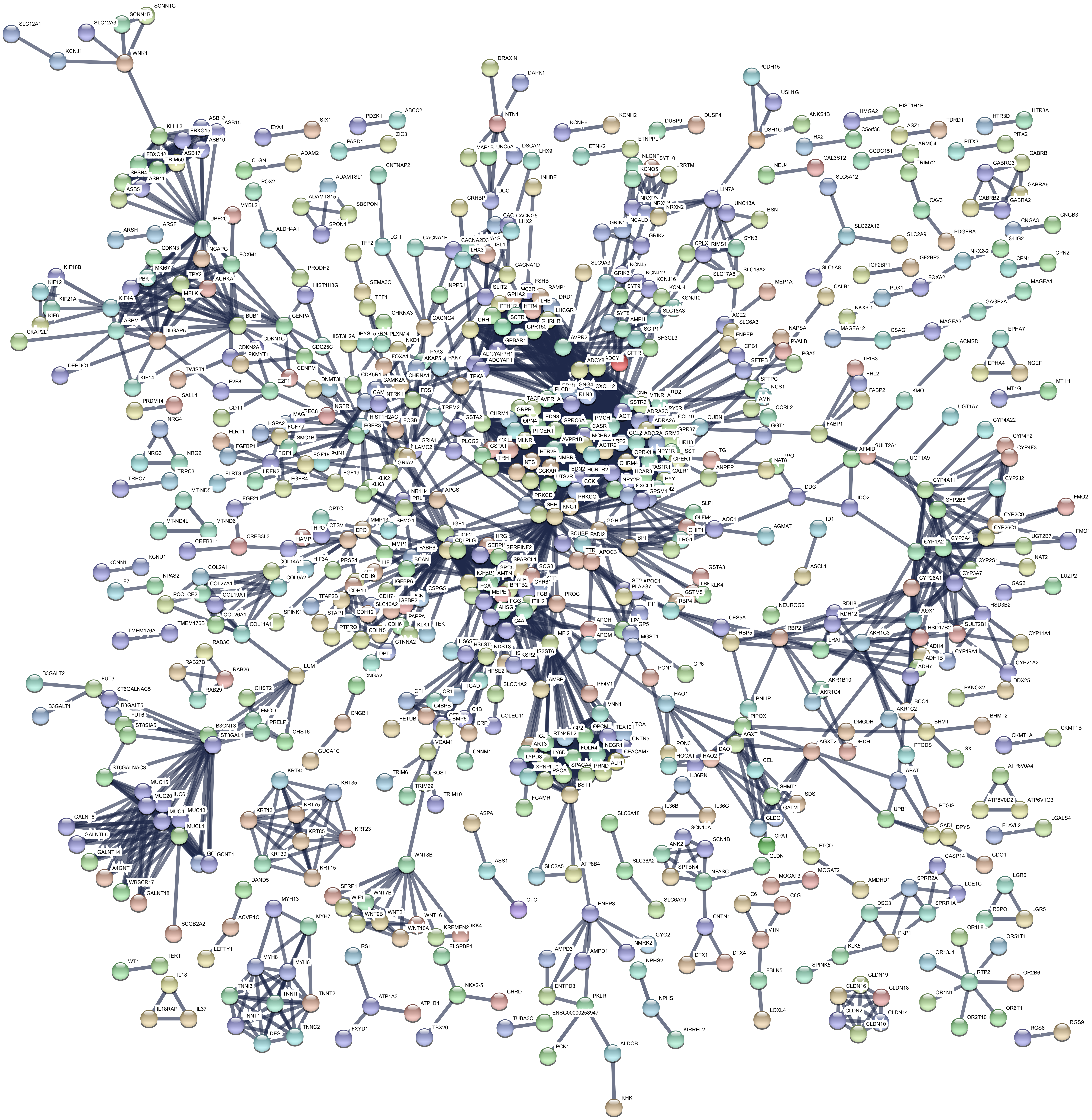

SLC12A1
KCNJ1
SLC12A3
SCNN1B
SCNN1G
WNK4
KLHL3
ASB15
ASB10
FBXO15
ASB17
FBXO40
TRIM50
SPSB4
ASB11
ASB5
UBE2C
ARSH
ARSF
CDKN3
NCAPG
FOXM1
MKI67
PBK
TPX2
AURKA
MELK
KIF18B
KIF12
KIF21A
KIF4A
KIF6
CKAP2L
ASPM
DLGAP5
BUB1
CENPA
CDKN1C
CDKN2A
PKMYT1
CDC25C
CENPM
DEPDC1
KIF14
TWIST1
E2F8
E2F1
DNMT3L
PRDM14
SALL4
NRG4
CDT1
FLRT1
FGFBP1
NRG3
NRG2
TRPC3
TRPC7
FLRT3
LRFN2
FGFR4
FGF21
MT-ND5
MT-ND4L
MT-ND6
CREB3L1
CREB3L3
KCNN1
KCNU1
F7
NPAS2
COL2A1
COL27A1
COL9A2
COL19A1
PCOLCE2
COL26A1
COL11A1
TMEM176B
TMEM176A
RAB3C
RAB27B
RAB26
RAB29
B3GALT2
FUT3
ST6GALNAC5
B3GALT5
B3GALT1
FUT6
ST8SIA5
B3GNT3
ST3GAL1
ST6GALNAC3
MUC15
MUC20
MUC4
MUC13
MUCL1
GALNT6
GALNTL6
GALNT14
A4GNT
WBSCR17
GALNT18
GCNT1
SCGB2A2
DAND5
ACVR1C
LEFTY1
WT1
TERT
IL18
IL18RAP
IL37
MYH13
MYH7
MYH8
MYH6
TNNI3
TNNI1
TNNT2
TNNT1
DES
TNNC2
RS1
ATP1A3
ATP1B4
FXYD1
TBX20
NKX2-5
CHRD
WNT8B
SFRP1
WIF1
WNT7B
WNT9B
WNT2
WNT16
WNT10A
KREMEN2
ELSPBP1
TRIM6
TRIM29
TRIM10
SOST
VCAM1
KRT40
KRT35
KRT13
KRT75
KRT85
KRT23
KRT39
KRT15
CHST2
FMOD
PRELP
CHST6
GUCA1C
LUM
CNGA2
CNGB1
DPT
EYA4
SIX1
PDZK1
ABCC2
PASD1
ZIC3
CLGN
ADAM2
ADAMTSL1
CNTNAP2
POX2
ADAMTS15
SBSPON
SPON1
MYBL2
ALDH4A1
PRODH2
HIST1H3G
TFF2
LGI1
SEMA3C
TFF1
CHRNA3
HIST3H2A
DPYSL5
PLXNA4
NKD1
FOXA1
PAK3
AKAP5
PAK7
CDK5R1
CAMK2A
CHRNA1
ITPKA
CAM
NTRK1
FOS
NGFR
HIST1H2AC
FOSB
FGFR3
HSPA2
MAG
FGF7
SMC1B
FGF1
FGF18
FGF19
GRIA1
LAMC2
GRIA2
KLK2
KLK3
NR1H4
PRL
APCS
GSTA2
TREM2
PLCG2
CHRM1
GSTA1
TRH
OPTC
THPO
CTSV
EPO
HAMP
MMP13
SEMG1
HIF3A
COL14A1
PRSS1
MMP1
LIF
FABP6
BCAN
IGF1
IGF2
PLG
CDH9
CDH10
IGFBP6
CSPG5
TFAP2B
CDH7
SLC10A2
CDH12
IGFBP2
SPINK1
STAP1
PAPPA
CDH6
PTPRO
CDH15
KLK1
TEK
CTNNA2
DRAXIN
DAPK1
NTN1
MAP1B
UNC5A
DSCAM
LHX9
INHBE
DCC
CRHBP
CACNG5
LHX2
CACNA1E
CACNA2D3
LHX3
ISL1
CACNA1D
CACNG4
INPP5J
SLIT2
MC3R
RAMP1
FSHB
GPHA2
DRD1
LHB
PTH1R
HTR4
LHCGR
CRH
SCTR
GHRHR
GPR150
GPBAR1
ADCYAP1
ADCYAP1R1
AVPR2
ADCY8
ADCY1
CFTR
PLCB1
GNG4
RLN3
CXCL12
TACR
AVPR1A
GRPR
EDN3
GPRC6A
PMCH
CASR
OPN4
PTGER1
AVPR1B
MLNR
HTR2B
NMBR
NTS
CCKAR
EDN2
HCRTR2
UTS2R
CCK
PRKCD
PRKCQ
SHH
KNG1
CHRM4
HCAR3
NPY2R
CXCL1
GPSM1
NPY1R
TAS1R1
GALR1
PYY
AGT
MCHR2
AGTR2
OPRK1
CCL2
ADORA
GRM2
HRH3
SST
GPER1
GPR37
ADRA2A
ADRA2C
CCL19
SSTR3
MTNR1A
CNR
NCS1
AMN
CCRL2
CUBN
GGT1
TG
TPO
ANPEP
NAT8
SFTPB
SFTPC
ENPEP
CPB1
PGA5
PVALB
NAPSA
SLC6A3
ACE2
MEP1A
KCNH6
KCNH2
ETNK2
ETNPPL
DUSP9
DUSP4
NLGN
SYT10
KCNQ5
LRRTM1
NRXN2
NCALD
GRIK1
GRIK2
GRIK3
KCNJ5
KCNJ12
KCNJ16
KCNJ4
KCNJ10
SLC9A3
SYT9
SYT8
AMPH
SLC18A3
SGIP1
SH3GL3
LIN7A
UNC13A
BSN
CPLX
RIMS1
SYN3
SLC17A8
SLC18A2
PCDH15
USH1G
USH1C
ANKS4B
HMGA2
HIST1H1E
IRX2
C5orf38
ASZ1
TDRD1
ARMC4
NEU4
GAL3ST2
CCDC151
TRIM72
SLC5A12
CAV3
SLC22A12
SLC2A9
PDGFRA
SLC5A8
CALB1
IGF2BP1
IGF2BP3
PDX1
FOXA2
NKX6-1
MAGEA12
CSAG1
MAGEA3
GAGE2A
MAGEA1
HTR3D
HTR3A
PITX3
PITX2
GABRB1
GABRG3
GABRB2
GABRA2
GABRA6
CNGA3
CNGB3
OLIG2
NKX2-2
CPN1
CPN2
EPHA7
EPHA4
NGEF
TRIB3
FHL2
FABP2
FABP1
ACMSD
KMO
MT1G
MT1H
UGT1A7
CYP4A22
CYP4F2
CYP4F3
SULT2A1
AFMID
UGT1A9
DDC
IDO2
CYP4A11
CYP2B6
CYP2J2
FMO2
FMO1
CYP2C9
CYP26C1
UGT2B7
CYP1A2
CYP3A4
NAT2
CYP2S1
CYP26A1
CYP3A7
GAS2
LUZP2
HSD3B2
RDH8
RDH12
AOX1
SULT2B1
CYP11A1
LRAT
AKR1C3
HSD17B2
ADH4
ADH1B
CYP19A1
CYP21A2
ADH7
DDX25
AKR1B10
AKR1C4
PKNOX2
BCO1
AKR1C2
BHMT
BHMT2
CKMT1A
CKMT1B
PTGDS
ISX
ABAT
UPB1
PTGIS
ATP6V0A4
ATP6V0D2
ATP6V1G3
ELAVL2
LGALS4
GADL
DPYS
CDO1
CASP14
SPRR2A
LCE1C
SPRR1A
LGR6
RSPO1
LGR5
DSC3
PKP1
KLK5
SPINK5
OR1L8
OR51T1
OR13J1
RTP2
OR2B6
OR1N1
OR6T1
OR2T10
RGS6
RGS9
CLDN19
CLDN16
CLDN18
CLDN2
CLDN14
CLDN10
ID1
ASCL1
NEUROG2
AGMAT
SLPI
OLFM4
CHIT1
AOC1
LRG1
BPI
GGH
PADI2
SCUBE
TTR
APOC3
GSTA3
GSTM5
KLK4
RBP4
F11
PLA2G7
APOC1
CES5A
RBP5
RBP2
SERPINF2
SPARCL1
SCG3
IGFBP1
AMTN
FGA
BPIFB2
CYR61
MEPE
FGB
FGG
ITIH2
AHSG
PROC
C4A
APOH
APOM
LPA
GP5
MFI2
MGST1
PON1
GP6
HAO1
PON3
HOGA1
HAO2
DAO
PIPOX
AGXT
AGXT2
PNLIP
DMGDH
DHDH
CEL
SHMT1
SDS
GATM
GLDC
CPA1
GLDN
FTCD
MOGAT3
MOGAT2
AMDHD1
C6
C8G
VTN
FBLN5
LOXL4
HS6ST2
NDST3
KSR2
HS3ST6
HPSE2
SLCO1A2
COLEC11
CFI
CR1
ITGAD
C4BPB
C4B
BMP6
CRP
CNNM1
FETUB
PF4V1
AMBP
VNN1
TEX101
TOA
CNTN5
CEACAM7
NEGR1
FOLR4
OPCML
RTN4RL2
ART3
LY6D
LYPD8
XPNPEP2
PSCA
SPACA4
PRND
ALPI
BST1
FCAMR
SLC2A5
ATP8B4
ENPP3
SLC6A18
SLC36A2
SLC6A19
IL36RN
IL36B
IL36G
SCN10A
ANK2
SCN1B
SPTBN4
NFASC
CNTN1
DTX1
DTX4
ASPA
ASS1
OTC
AMPD3
AMPD1
ENTPD3
PKLR
ENSG00000258947
NMRK2
GYG2
NPHS2
NPHS1
KIRREL2
TUBA3C
PCK1
ALDOB
KHK
